# The East–West Gap in Pay Fairness Perceptions Converged Between 2009 and 2023

**DOI:** 10.1007/s11577-026-01086-5

**Published:** 2026-06-10

**Authors:** Jule Adriaans, Carsten Sauer

**Affiliations:** Faculty of Sociology, Bielefeld University, Bielefeld, Germany

**Keywords:** Justice of gross earnings, Regional differences, Time series, SOEP, Lohngerechtigkeit, Regionale Unterschiede, Zeitreihe, SOEP

## Abstract

This study investigates regional differences in pay fairness perceptions among employees in eastern and western Germany from 2009 to 2023. Using longitudinal data from the Socio-Economic Panel study, the analysis reveals substantial initial disparities: Employees in eastern Germany perceive their gross earnings as more unfair than employees in western Germany do. Over time, however, these regional differences diminish. Analyses stratified by wage deciles show that this convergence in pay fairness is primarily driven by a decrease in unfairness perceptions among low-wage employees in eastern Germany. This development, in turn, is driven by a narrowing East–West gap in actual earnings among low-wage employees. The finding of converging pay fairness perceptions in eastern and western Germany remains robust after adjusting for individual differences (gender, age, education, labor market experience, immigration background) and structural factors (economic area, public sector, organizational size), underscoring that there has been a true convergence of perceptions over time. While this development is cause for optimism, the overall convergence is driven not solely by employees in eastern Germany improving in terms of pay fairness but also by employees in western Germany showing an overall increase in perceived unfairness.

**Supplementary Information:**

The online version of this article (https://doi.org/10.1007/s11577-026-01086-5) contains supplementary material, which is available to authorized users.

## Abstract (de)

Diese Studie untersucht regionale Unterschiede in der Wahrnehmung der Lohngerechtigkeit unter Beschäftigten in Ost- und Westdeutschland im Zeitraum von 2009 bis 2023. Anhand von Längsschnittdaten des Sozio-oekonomischen Panels zeigt die Analyse zunächst erhebliche Unterschiede: Beschäftigte in Ostdeutschland empfinden ihre Bruttoeinkommen als ungerechter als jene in Westdeutschland. Im Laufe der Zeit verringern sich diese regionalen Unterschiede jedoch. Nach Lohndezilen aufgeschlüsselte Analysen zeigen, dass diese Annäherung in der Wahrnehmung der Lohngerechtigkeit in erster Linie auf einen Rückgang der Wahrnehmung von Ungerechtigkeit bei Niedriglohnbeschäftigten in Ostdeutschland zurückzuführen ist. Diese Entwicklung wiederum wird durch ein sich verringerndes Ost-West-Gefälle in den tatsächlichen Verdiensten der Niedriglohnbeschäftigten angetrieben. Die beobachtete Angleichung zwischen Ost- und Westdeutschland in der Wahrnehmung der Lohngerechtigkeit bleibt auch nach Kontrolle individueller (Geschlecht, Alter, Bildung, Berufserfahrung, Migrationshintergrund) und struktureller Faktoren (Wirtschaftsraum, öffentlicher Sektor, Unternehmensgröße) bestehen. Dies unterstreicht eine echte Konvergenz der Wahrnehmungen im Laufe der Zeit. Zwar gibt diese Entwicklung grundsätzlich Anlass zu Optimismus, jedoch wird die Annäherung nicht allein durch eine Verbesserung der Lohngerechtigkeit in Ostdeutschland getrieben, sondern auch durch eine Zunahme an wahrgenommener Ungerechtigkeit unter Beschäftigten in Westdeutschland.

## Introduction

More than 30 years after reunification, sizeable structural differences remain between eastern and western Germany. As of 2023, the average hourly wage in eastern Germany was approximately 17% lower than in western Germany (Destatis 2025a). Moreover, there are persistent structural differences in economic strength, as well as in the composition of industries and the workforce (Brenke 2014; Brüll and Gathmann 2020; Krause 2019). More generally, eastern Germany continues to be characterized by lower population density, higher median age, and lower shares of immigrants compared to western Germany (Destatis 2025b; Krause 2019).

East–West gaps, however, are not limited to such structural factors. For example, over the 30 years after reunification (1990–2020), average life satisfaction was consistently lower in eastern Germany than in western Germany (Krause 2019). Eastern Germans were also more worried about their economic condition than their western German counterparts were (Luhmann et al. 2010). People in eastern Germany have also reported lower levels of political and institutional trust (Braun and Trüdinger 2022; Campbell 2012). Pickel and Pickel (2023) further demonstrate that although respondents in eastern Germany are no less supportive of democracy in general, they are less satisfied with the current state of democracy. They argue that this East–West gap cannot be explained by objective socioeconomic disadvantage in the East but rather by feelings of deprivation and a sense of lacking recognition. Indeed, past research has documented higher unfairness perceptions among workers in eastern Germany (Liebig et al. 2014; Sauer et al. 2016; Wegener and Liebig 2010), with higher overall perceptions of injustice in eastern Germany compared to western Germany (Jasso 1999). Ideological differences in the principles that should guide the allocation of goods and burdens in a society have also been observed: Respondents in eastern Germany expressed greater support for equality of outcomes than those in western Germany (Hülle et al. 2018; Wegener and Liebig 2000, 2010). Such fairness attitudes relate directly to the legitimacy of economic inequality (Jasso 1999; Liebig and Sauer 2016). If workers in eastern Germany are indeed experiencing higher levels of perceived unfairness—as suggested by previous research—this not only exposes them to the adverse outcomes associated with unfairness (e.g., Adriaans 2023; Schnaudt et al. 2021; Schunck et al. 2015) but also calls into question the legitimacy of the economic conditions they experience, potentially contributing to regional conflicts. A convergence in pay fairness perceptions among workers in eastern and western Germany would, however, suggest common ground regarding the legitimation of economic inequality.

While much evidence documents that meaningful regional disparities have persisted between eastern and western Germany after reunification, recent evidence suggests that East–West gaps have narrowed in various domains. The gaps in satisfaction regarding health, jobs, and income, as well as the gap in mental health, have narrowed (Beckmannshagen et al. 2023; Buchinger et al. 2024). Investigating trends in institutional trust between 1984 and 2018, Campbell (2023) reports that regional gaps are decreasing over time. However, Faus and Storks (2019) conclude that for young Germans born after reunification, being from eastern Germany is part of their identity, whereas this is not the case for respondents from western Germany. Mau et al. (2024) also find that while perceptions of East–West conflicts and the East as “other” have declined among young western Germans, this is not the case among young eastern Germans.

The question of whether fundamental East–West differences—in attitudes, ideology, and living conditions—are on a trajectory of convergence, stability, or continued polarization is controversially debated (Mau 2024; Oschmann 2023; Pollack 2020). In this descriptive research note, we aim to provide new evidence on the development of regional differences in fairness perceptions by tracing pay unfairness—a key indicator of perceived deprivation—over a 15-year period. We rely on data from the Socio-Economic Panel study (SOEP) from 2009 to 2023, with information on pay fairness queried every other year; SOEP data offer the longest available time series of pay fairness evaluations in Germany, allowing us to identify long-lasting trends in how perceptions of pay fairness developed in eastern and western Germany. In the following, we first provide a brief theoretical background on empirical justice research in sociology and the conceptual framework used in this note. Next, we describe the data and variables we rely on and then present evidence of a narrowing East–West gap in pay fairness perceptions over time. We conclude this note with a brief discussion of our findings.

## Theoretical Background

Questions of fairness have a long intellectual tradition. In practical philosophy, the central aim is normative: to justify rules and principles specifying what people ought to receive and how they ought to be treated. From classical accounts of distributive justice (e.g., proportionality between merit and reward) to modern theories that stress equal moral worth, rights, or capabilities, normative approaches ask what a just distribution is and how it can be defended as legitimate. Sociology of justice takes an empirical starting point. Rather than adjudicating which allocation rule is morally acceptable, it asks what people perceive as fair, how such standards are formed, and what consequences follow when expectations are violated (Liebig and Sauer 2016). This research program—systematically developed over roughly the last five decades—treats fairness perceptions as a core element of social order because they link individual evaluations to compliance, cooperation, and legitimacy in institutions (Sabbagh and Schmitt 2016). Individuals hold situation-specific beliefs about what constitutes fair treatment and a just reward; when outcomes or procedures deviate from these standards, they are experienced as unfair (Walster et al. 1973). A key insight of this literature is that fairness perceptions are subjective, yet not arbitrary. The present research note focuses on the sense of distributive justice, i.e., the fairness of outcomes, in the labor market, namely the perceived fairness of one’s own earnings—an evaluation that has been repeatedly linked to consequences extending beyond the workplace. Workers who evaluate their pay as unfair report lower life satisfaction (Adriaans 2023), poorer health (Schunck et al. 2015), and lower political trust (Schnaudt et al. 2021).

In the context of earnings, fairness standards are often theorized in terms of social exchange and reciprocity. The norm of reciprocity implies that contributions should be met with commensurate returns; higher perceived inputs—such as effort, performance, responsibility, or skills—are expected to be rewarded by higher pay (Adriaans et al. 2023). This logic underpins the classic perspective of equity theory (Adams 1965), according to which individuals evaluate whether the ratio of their inputs to outcomes is proportional to that of relevant others. A second central mechanism is social comparison. People do not evaluate their earnings in isolation; instead, they use comparison information to infer what is typical, deserved, or legitimate. Work-related comparison targets—colleagues in the same workplace, workers in similar occupations, or people with comparable qualifications—are especially influential because they provide salient benchmarks for what “someone like me” should earn (Bygren 2004; Adriaans et al. 2023; Eisnecker and Adriaans 2024). Comparison processes are therefore not only psychological but also socially structured (Sauer and Adriaans 2025). Labor market segmentation, workplace visibility, regional differences, and institutionalized wage-setting practices determine which reference groups are available and credible.

Once individuals hold a standard of what they should receive, unfairness arises when the actual reward deviates from that standard. If actual earnings fall short of the fair standard, individuals experience unjust underreward. If actual earnings exceed what is considered fair, individuals experience unjust overreward. Importantly, empirical justice research emphasizes that the magnitude of deviation matters: Larger discrepancies between “what I get” and “what I deserve” generate stronger injustice experiences and more pronounced downstream consequences (Jasso 1978; Jasso et al. 2016).

Although justice evaluations are subjective, they are shaped by institutional and cultural contexts that influence (a) which fairness principles are viewed as legitimate (e.g., equity, equality, need), (b) which comparison targets are salient, and (c) how earnings are interpreted as signals of merit, recognition, or social status. This is crucial for understanding variation across social groups and over time: Fairness perceptions respond not only to individual experiences (e.g., raises, job changes) but also to shifting labor market conditions and the institutional organization of wage-setting that structures both rewards and the benchmarks used to evaluate them (e.g., collectively bargained pay scales, wage transparency, and the perceived legitimacy of market outcomes).

Against this backdrop, differences in pay fairness perceptions across contexts (such as eastern and western Germany) can be interpreted as differences in reference standards, comparison structures, and institutional embedding of wage formation. Changing fairness perceptions over time therefore reflect changes in the social and institutional environments that shape “what is fair” in labor market reward allocations.

## Data, Variables, Analysis

### Data

This study is based on the SOEP, version 40, released in 2025 (Goebel 2025, https://doi.org/10.5684/soep.core.v40eu). The SOEP is a longitudinal household survey conducted annually since 1984 by the German Institute for Economic Research (DIW Berlin). It provides micro-level data on individuals, households, and their socioeconomic conditions in Germany, making it particularly suitable for examining trends in labor market outcomes and attitudes over time. The data used in this study cover the period from 2009 to 2023, allowing an analysis of recent developments and long-term changes in regional differences in pay fairness between eastern and western Germany. During this period, respondents had been asked about the fairness of their earnings on a biannual basis.

We restricted the analytical sample to respondents in paid employment aged 18–65 years with valid information on their perceived fairness and hourly wages (*N* = 75,583). Follow-up analyses that account for differences in human capital variables (gender, age, education, work experience), immigration background, firm size, economic area, and public sector rely on a reduced sample size after listwise deletion on covariates (*N* = 66,600). See Online Supplement Table A1 for a complete overview of observations per year.

### Variables

Our focal variable is the *fairness perception of one’s hourly gross pay*. Following justice evaluation theory, fairness perceptions are defined as the logarithmic ratio between actual and subjectively fair pay, calculated as follows (Jasso 1978; Jasso et al. 2016; Moya and Adriaans 2024):

$$J=\ln \left(\frac{\textit{Actual}\,\textit{pay}}{\textit{Fairpay}}\right)$$

If the ratio between actual and fair pay is balanced, the fairness perception *J* is zero, capturing the point of perfect fairness. Positive values of *J* indicate unfair overreward (actual pay is higher than fair pay). The perception of overreward, however, is rare (Adriaans 2023; Sauer and Valet 2013), with fewer than 3% of the sample reporting unfair overreward. Negative values instead indicate unfair underreward; that is, respondents perceive themselves as receiving less than what they would deem fair, with larger absolute values indicating a stronger sense of unfairness. To ease interpretation, we multiplied *J* by −1, so increasing values indicate more intense unfair underreward:

$$\textit{Unfairness}=-1\times J$$

The perceived unfairness of pay is based on two quantities: actual pay and fair pay. Respondents provided information on their monthly gross pay and their monthly fair pay. The latter information was queried in a two-step process. First, respondents were asked whether they considered their pay fair or not.^1^ For those respondents who evaluated their pay as fair, fair pay equals actual pay. Those who stated that their pay was unfair were asked, “How high would your gross income have to be in order to be just?” We then relied on respondents’ reported weekly working hours to convert monthly pay—both actual and fair—into hourly wages. Extreme outliers, i.e., the top and bottom 1% of the hourly wage distribution, were trimmed from the analysis.^2^

Our primary focus is on differences in unfairness perceptions between respondents from eastern and western Germany. A binary indicator captured whether respondents were currently residing within the former German Democratic Republic (1 = East) or within the borders of the Federal Republic of Germany as of 1989 (0 = West). About 17% of all respondents in the analytical sample lived in eastern Germany.

*Covariates: *In addition to showing aggregate differences in pay fairness between eastern and western Germany over time, we investigated regional differences while accounting for compositional differences in the eastern and western German workforce and labor market. We controlled for *education* using the Comparative Analysis of Social Mobility in Industrial Nations (CASMIN) classification.^3^ We further accounted for *relative work experience*. We captured relative work experience as the ratio of actual work experience—measured as the sum of full-time and part-time work experience, where part-time work is weighted by 0.5—to potential work experience—captured by respondent age, subtracting years of education and 6 years because we assume that potential work experience starts only after the completion of full-time education.^4^ We included a squared term as well to account for potential nonlinear effects of experience. Using relative work experience allows us to further account for respondent *age* and its squared term. Accounting for age and work experience simultaneously is essential, as the age structure and employment biographies differ notably between the two regions (Krause 2019). Finally, we included information on respondent gender (1 = female, 0 = male) and *immigration background* (1 = no immigration background, 2 = direct immigration background, 3 = indirect immigration background) to account for East–West differences in workforce composition.

In addition, we controlled for structural differences in the labor market. We controlled for *firm size* in four categories: < 20 employees, 20 to < 200 employees, 200 to < 2000 employees, and > 2000 employees. We also adjusted for 13 *economic areas* in which employees work.^5^ Finally, we included a dummy indicator of whether respondents were currently working in the *public sector* (1).

### Analysis

This paper aims to trace the trajectory of pay fairness perceptions in eastern and western Germany over time. To do this, we provide descriptive analyses of aggregate fairness evaluations over time, by region, and by wage decile to capture where changes have occurred in the earnings distribution.

In a final step, we investigated whether regional differences in pay fairness persisted after accounting for compositional differences between eastern and western Germany.

$$\textit{Unfairness}_{i}=\beta _{0}+\beta _{1}East_{i}+\gamma X_{i}+\delta Y_{i}+\varepsilon _{i}$$

We regressed the perceived unfairness of pay on an East–West dummy and the list of covariates described above, including both human capital (*X*_*i*_) and structural indicators (*J*_*i*_). *β*_1_ captures the East–West gap in unfairness perceptions, while adjusting for compositional differences between regions. We ran separate linear regression models for each survey year, with robust standard errors. All analyses employ cross-sectional weights provided by the SOEP.^6^

## Results

**Fig. 1:**
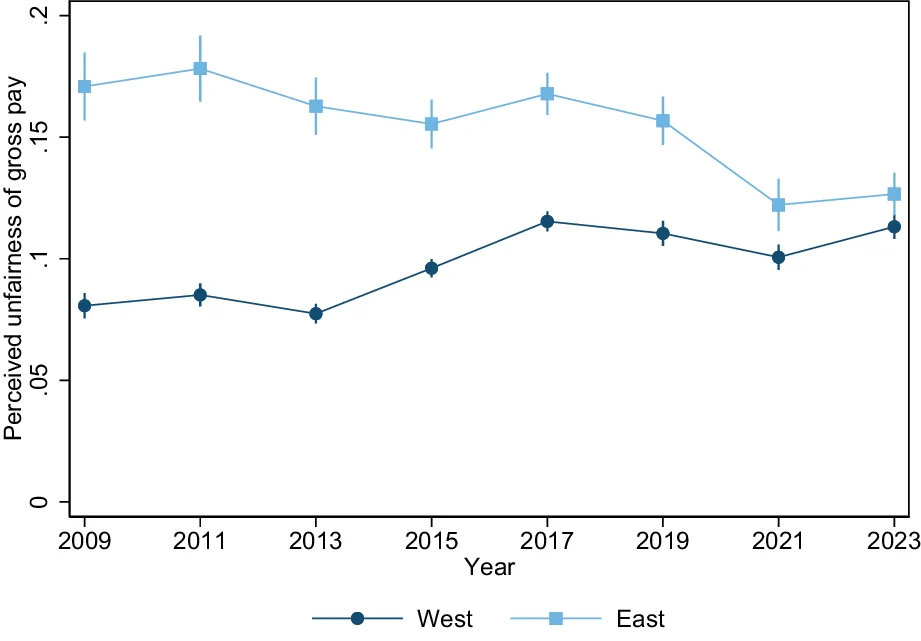
Trends in perceived unfairness of gross pay among employees in eastern and western Germany, 2009–2023. Mean values with 95% confidence intervals. A higher value indicates a greater perception of unfair underpayment. The data illustrate a gradual convergence between the two regions over time, driven by increasing perceived unfairness in the West and a decline in the East. Data: Socio-Economic Panel version 40

Fig. 1 shows the perceived unfairness of gross pay among employees in eastern and western Germany between 2009 and 2023. The data show a persistent regional difference over most of the observed period. Employees in eastern Germany consistently perceived their gross pay as more unfair compared to their counterparts in western Germany. However, the disparity between the two regions decreases over time. Specifically, the perceived unfairness of pay in western Germany rose gradually from approximately 0.08 in 2009 to around 0.11 by 2023. Thus, in western Germany, employees, on average, perceived their pay as increasingly unfair. Conversely, the perceived unfairness of pay in eastern Germany remained relatively stable, ranging between 0.16 and 0.18 from 2009 to 2017, after which it shows a moderate downward trend, reaching 0.13 in 2023. Overall, the change between 2009 and 2023 is somewhat more pronounced in eastern Germany (with unfairness decreasing by 0.21 standard deviation) than in western Germany (with unfairness increasing by 0.15 standard deviation). As a result of these opposing trends, the gap in perceived pay unfairness between eastern and western Germany narrowed significantly over the studied period, indicating convergence in unfairness perceptions among employees from both regions. The last wave, 2023, marks the first year in the studied period in which there was no statistically significant gap in unfairness perceptions between east and western Germany.

**Fig. 2:**
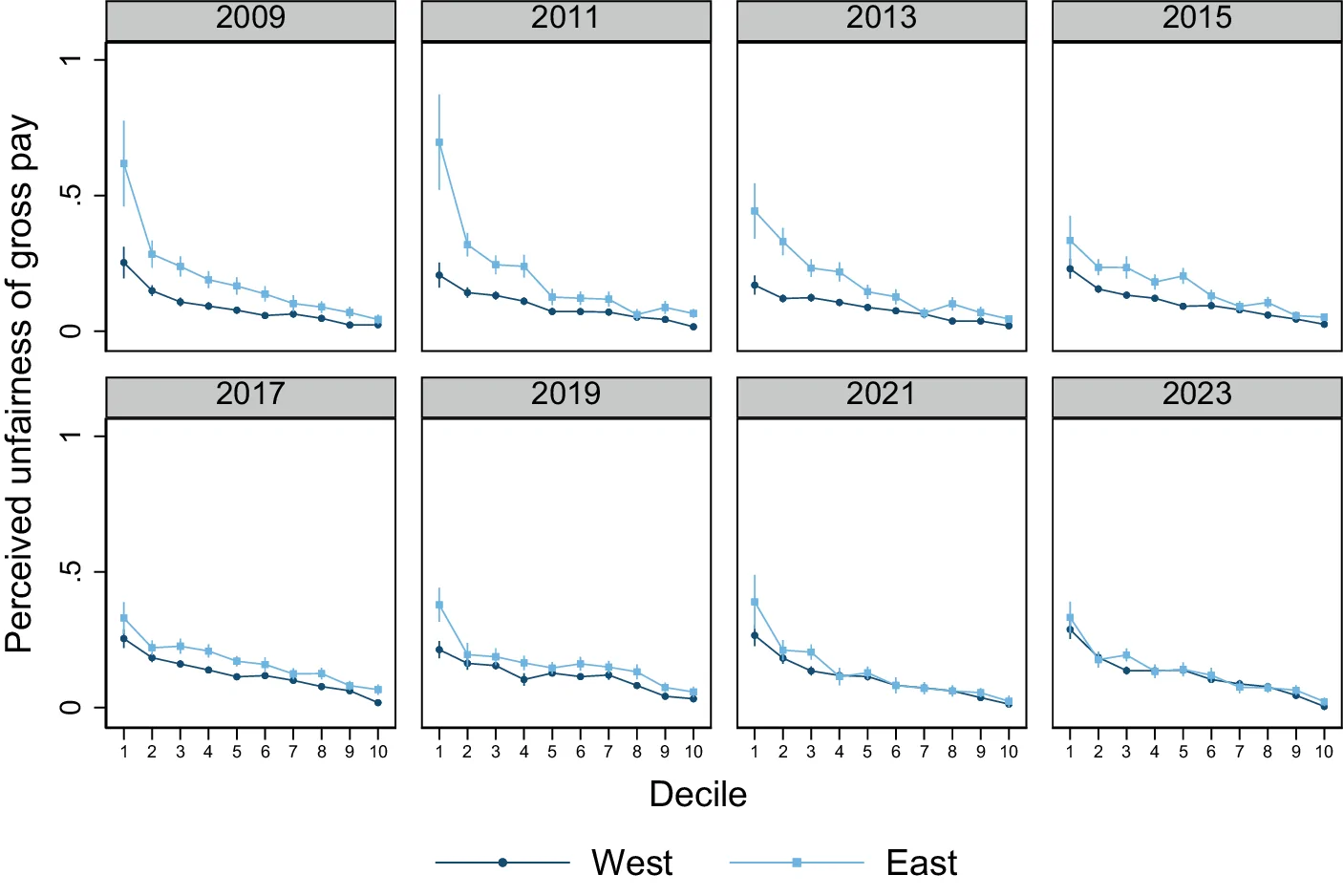
Perceived unfairness of gross pay among employees in eastern and western Germany by wage decile from 2009 to 2023. Each subgraph represents one survey year, showing mean values with 95% confidence intervals. Higher values indicate greater perceived unfairness. The figure reveals pronounced regional disparities primarily in lower- and middle-wage deciles, with employees in eastern Germany reporting higher unfairness perceptions (i.e., perceived underpayment). Over time, these regional differences narrowed, driven mainly by declining perceptions of unfairness among lower-wage employees in eastern Germany. Data: Socio-Economic Panel version 40

The sense of unfairness concerning one’s earnings is directly related to the earnings one receives (Jasso 1978). Indeed, past research has shown that pay unfairness is more pronounced at the lower end of the earnings distribution and that perceptions of unfairness decrease as earnings increase (e.g., Adriaans and Targa 2023; Sauer et al. 2016). To capture whether the aggregate patterns observed in Fig. 1 differ across the wage distribution, Fig. 2 shows the perceived unfairness of gross pay among employees in eastern and western Germany from 2009 to 2023, stratified by region-specific hourly wage deciles.^7^ Each subgraph corresponds to a specific survey year in which fairness perceptions were measured (2009, 2011, 2013, 2015, 2017, 2019, 2021, and 2023), showing how perceptions of pay unfairness vary systematically across the wage distribution. Across all time points, a consistent pattern emerges: Perceived unfairness decreases as the position in the hourly earnings distribution increases. This negative gradient suggests that employees in lower-wage deciles consistently perceive greater pay unfairness compared to those in higher-income groups.

Beyond this overall trend, regional differences between eastern and western Germany are visible across the wage distribution, particularly in the first half of the studied period (2009–2015). In the lower- and middle-wage deciles (1–6), employees in eastern Germany reported substantially higher levels of perceived unfairness of pay than their western German counterparts. This pattern is particularly pronounced in 2009, 2011, and 2013. However, in higher-wage deciles (7–10), the differences between East and West are much less pronounced and are typically statistically negligible.

Importantly, the regional disparities in how respondents in low- and middle-wage deciles evaluated their pay decreased steadily over time. By 2023, the two lines mapping the intensity of unfairness across the wage distribution in eastern and western Germany are almost completely aligned. Thus, the convergence in aggregate unfairness perceptions in eastern and western Germany over the years, observed above, was primarily driven by decreasing perceptions of unfairness among eastern German employees in lower-income deciles, alongside a slight increase or stability in unfairness perceptions among western German employees across similar deciles.

Overall, Fig. 2 shows the complexity underlying the convergence in pay fairness perceptions between eastern and western Germany. It highlights that the overall trend toward convergence observed in Fig. 1 resulted primarily from changes among lower-income employees, where regional differences were initially most pronounced but have subsequently narrowed.

**Fig. 3:**
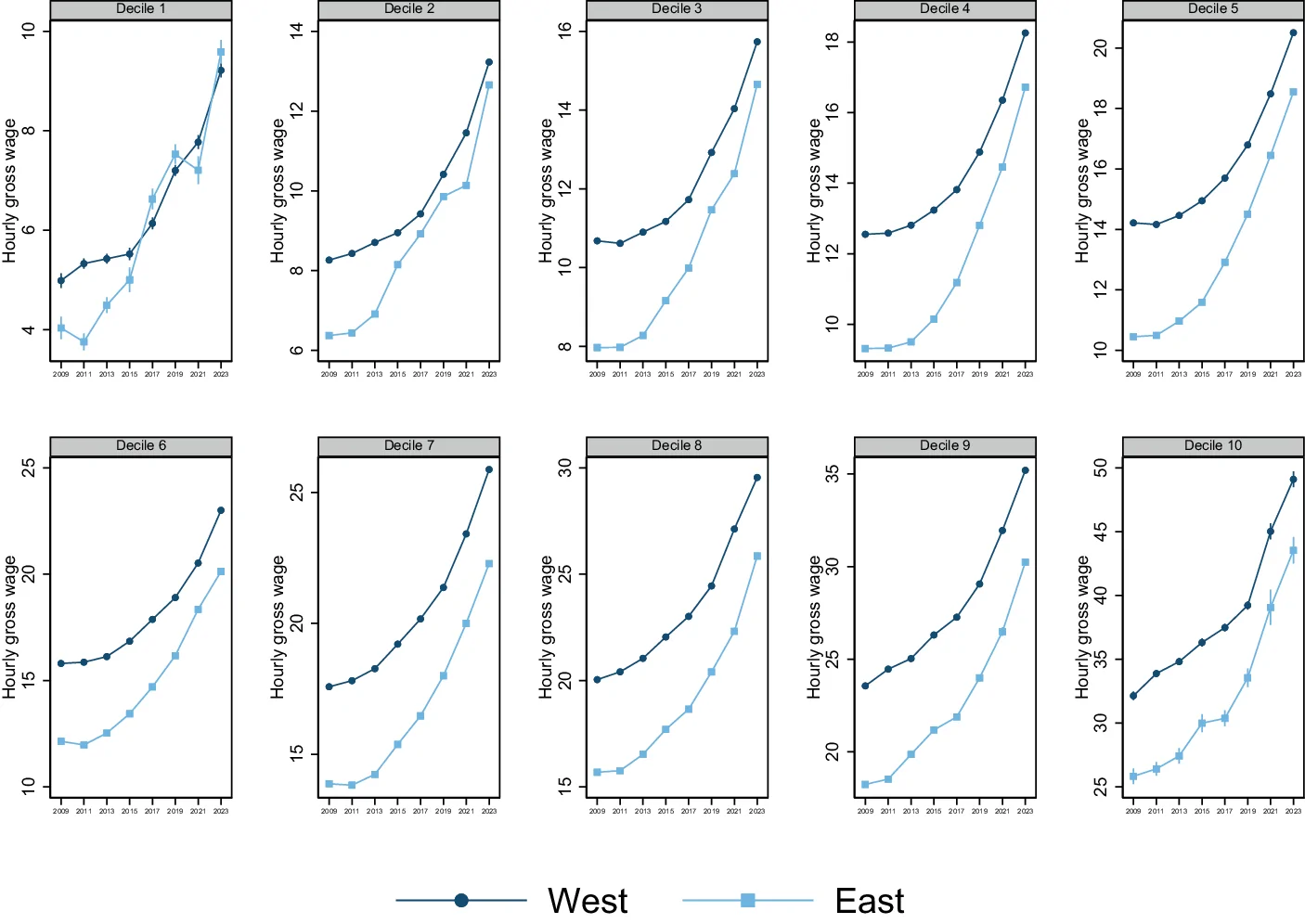
Nominal hourly gross wages of employees in eastern and western Germany by wage decile from 2009 to 2023. Each of the ten subgraphs represents a wage decile, ranging from the lowest (decile 1) to the highest (decile 10). The data illustrate regional disparities and differing growth dynamics across wage groups: Lower-wage deciles initially experienced different hourly wages but converged over time due to faster wage increases in eastern Germany, whereas middle- and higher-wage deciles exhibited consistently higher wages in western Germany throughout the period. This context helps explain the regional convergence observed in pay fairness perceptions, particularly within the lower-wage segments. Data: Socio-Economic Panel version 40

Fig. 1 and Fig. 2 show that perceptions of unfairness differ between eastern and western Germany but have converged over time, with lower-wage deciles driving this change. Given the close association between actual pay and perceptions of pay fairness, Fig. 3 shows changes in nominal hourly gross wages for employees in eastern and western Germany from 2009 to 2023 separately for each wage decile. The figure thus allows us to explore whether the East–West convergence in fairness is accompanied by an East–West convergence in low to medium wages. Fig. 3 comprises ten subgraphs, each representing a different decile, ordered from the lowest (1) to the highest (10). This detailed depiction allows for an in-depth analysis of regional earnings trajectories across the wage distribution.

Overall, a clear pattern emerges: Nominal hourly wages increased substantially in both regions and across all deciles throughout the entire observation period. However, regional disparities are evident in the magnitude and dynamics of these increases. In the lowest wage deciles (especially 1–3), the initial differences in hourly gross pay between eastern and western Germany were relatively large in 2009. However, the growth trajectories diverged notably afterward. Hourly gross wages in the lowest decile increased faster in eastern Germany after around 2013, resulting in a declining wage gap between East and West in decile 1. Remarkably, a similar pattern—characterized by faster wage growth in eastern Germany and, thus, a narrowing gap in nominal wages—also appears in deciles 2 to 5. Moving to the middle and upper deciles (6–10), regional disparities in hourly wages were pronounced initially. In these deciles, western German employees earned substantially higher hourly wages than their eastern German counterparts throughout the observation period. While earnings grew in both regions, the relative gap between East and West remained relatively stable over time, especially in deciles 7 and 8.

Taken together, Fig. 3 provides insights into the broader socioeconomic context underlying the convergence in perceptions of pay unfairness described earlier. Although nominal hourly wages increased across all earnings deciles and regions, eastern German employees in lower wage groups experienced faster earnings growth relative to western German employees after initial disparities in the early years. This might have contributed to persistently higher perceived unfairness among lower-wage employees in eastern Germany until recent years. These findings suggest that changes in pay fairness perceptions are closely tied to shifts in regional wage dynamics, especially within lower- and middle-income segments of the labor market. The regional convergence in perceived unfairness over the studied period appears partly driven by the relative improvements in earnings among lower-wage eastern German employees.

**Fig. 4:**
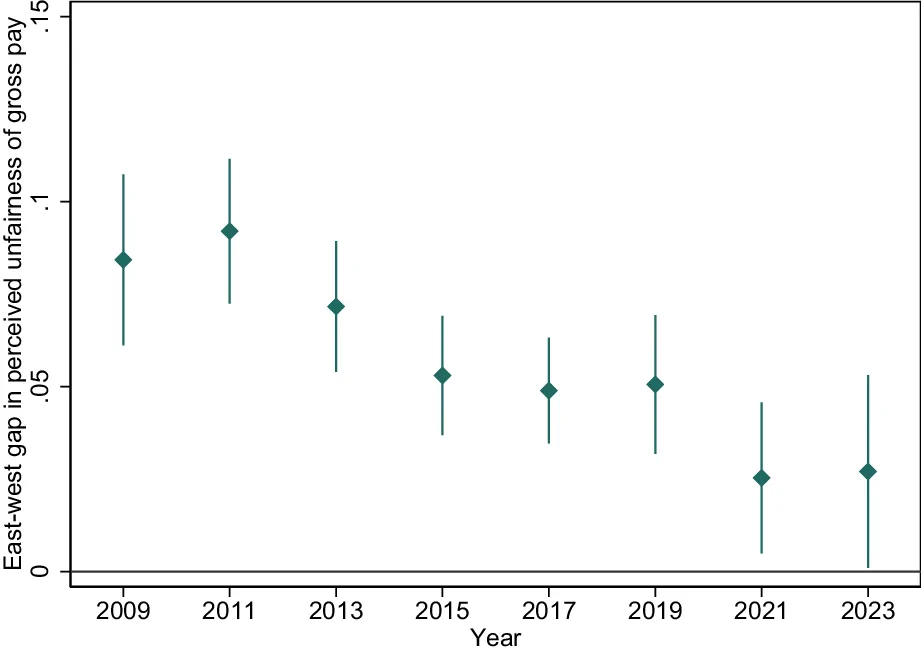
Adjusted East–West gap in perceived unfairness of gross pay among employees in Germany, 2009–2023. Estimates reflect regression results that control for individual human capital (gender, age, years of education, relative work experience, and immigration background) and structural characteristics (organizational size, economic area, and public sector). All models use listwise deletion for missing values on covariates. Point estimates are displayed with 95% confidence intervals. The results indicate a decline in regional disparities over time. Data: Socio-Economic Panel version 40

The descriptive results reported above do not account for compositional differences in individual and structural factors across regions. Therefore, in the last step of the analysis, we report the results of regression analyses by year. Fig. 4 shows the adjusted East–West gap in perceived unfairness of gross pay from 2009 to 2023, estimated using regression models that control for individual-level human capital and structural factors such as organizational size and economic area (see Online Supplement Table A2 for the full regression results). The plot displays point estimates with corresponding 95% confidence intervals, reflecting the magnitude and statistical uncertainty of regional disparities after accounting for these covariates.

The results reveal a downward trend in the adjusted East–West gap in perceptions of pay unfairness over the studied period. In 2009, employees in eastern Germany perceived their pay as being substantially more unfair than their western German counterparts did, even after relevant individual and structural factors were controlled for. This adjusted regional difference was about nine percentage points. Over subsequent years, the gap consistently and substantially narrowed. This pattern underscores the convergence in perceptions of pay fairness between eastern and western Germany. By 2023, the adjusted difference in perceived unfairness is, however, still statistically significant (*p* = 0.042). Overall, Fig. 4 complements the descriptive findings presented in previous figures by highlighting that the convergence of perceived pay unfairness between eastern and western Germany reflects a true convergence in perceptions that is independent of compositional differences in human capital and structural factors between eastern and western German employees.

## Discussion

Relying on survey data spanning 15 years, from 2009 to 2023, we traced recent trends and long-term developments in perceived pay fairness in Germany. We focused on regional differences between eastern and western Germany and found that although the perceived unfairness of pay was initially much more pronounced in eastern Germany, these regional disparities have narrowed considerably. In the last year of observation, 2023, there was no longer an East–West gap in average pay fairness. Subsequent analyses suggest that, first, the East–West gap was most pronounced in the lower half of the hourly wage distribution, and second, workers in eastern Germany in these wage groups saw significant improvements in their sense of unfairness regarding their pay. Follow-up analysis further highlighted that this group experienced faster nominal wage growth than their western German counterparts. The catch-up in wages among eastern German low-wage workers is likely related to the introduction of the national minimum wage in 2015. Indeed, previous literature documents that low-income workers in eastern Germany have particularly benefited from the minimum wage reform and that the East–West wage gap has narrowed as a result (Bossler and Schank 2023; Seils and Emmler 2020). The simultaneous decrease in actual wage gaps and regional disparities in perceptions of unfairness seems to support the view that East–West gaps in attitudes and perceptions will disappear as structural disparities dissolve (e.g., Wegener and Liebig 2000). It remains to be seen, however, whether the pattern of East–West convergence in pay fairness perception stabilizes over time. In our analyses, we focused on regional wage dynamics in shaping overall trends in perceived pay fairness. However, workers in Germany may compare themselves not only to others in paid work but also to the unemployed. Fig. A3 plots the region-specific unemployment rate next to the change in unfairness over time. While there is some resemblance in the downward trend in unemployment and in unfairness in eastern Germany between 2009 and 2021, pay fairness and unemployment in western Germany seem completely decoupled, suggesting that unemployment is not a major driver of changes in pay fairness among workers in Germany.

In any case, the improved pay fairness perceptions observed among workers in eastern Germany—particularly among low-wage workers—paint an optimistic picture of a decreasing sense of economic deprivation in eastern Germany and do not suggest increasing polarization among low-income individuals. At the same time, recent evidence suggests that East–West conflicts are still prevalent, and may even be intensifying, among young eastern Germans (Mau et al. 2024). Findings by Pickel and Pickel (2026) from the most recent federal election further suggest that support for the Alternative for Germany (AfD) party is driven by perceptions of collective, rather than personal, deprivation. Moreover, the overall pattern of convergence in perceived unfairness of pay is not driven just by improvements in the East but also by worsening perceptions of unfair pay in western Germany. It would therefore be overly optimistic to assume that the situation regarding the sense of fairness in Germany is improving more generally.

Finally, our analyses focused on the fairness of one’s wage. People, however, also form evaluations of all kinds of other rewards, the outcomes of others, and the principles that should guide the allocation of goods and burdens in a society (Liebig and Sauer 2016). Determining whether workers in eastern and western Germany fully converged on what they consider fair thus requires further research.

## Supplementary Information

Supplementary Material

## Data Availability

All analyses rely on the Scientific Use File of the Socio-Economic Panel study (release version 40, https://doi.org/10.5684/soep.core.v40eu). Researchers may apply for data access to the dataset underlying the analyses by submitting a user-contract application to the SOEP Research Data Center: (https://www.diw.de/en/diw_01.c.678568.en/research_data_center_soep.html). Full replication code is available here: https://osf.io/egpyh/

## Footnotes

1 The question format that filters respondents into a follow-up question on fair pay was changed in the 2015 wave. Before then, respondents were simply asked to state whether they felt their pay was fair or not (“yes” or “no”). Since 2015, respondents have been asked to evaluate the fairness of their pay on an 11-point scale ranging from unfairly low (−5) to unfairly high (+5), where 0 indicates “fair” pay. Adriaans et al. (2022) show that *J* is robust to this change in methodology.

2 Figure A1 in the Online Supplement illustrates that our main finding of a declining East–West gap in unfairness, with increasing unfairness perceptions in the West and decreasing unfairness perceptions in the East, is generally robust to this exclusion criteria. A notable deviation between Fig. A1 and Fig. 1 arises for the most recent observation in 2023, where the figure including extreme outliers suggests an increase in unfairness among eastern German workers from 2021 to 2023. Extreme outliers, by definition, affect only a very small share of the sample, and the perceived unfairness of own—actual and fair—hourly pay combines information from multiple open-response formats, making it particularly prone to be affected by measurement error due to implausible answers (e.g., monthly gross earnings of 90,000 euros and more). Another issue, potentially arising from using information from open-format questions on income and working hours is that such questions, especially the former, are prone to item nonresponse (e.g., Valet et al. 2019). Accordingly, the share of missing information for our key indicator, unfairness of gross pay, is relatively high, but it is comparable for eastern (17.71%) and western Germany (18.1%).

3 Education is captured in ten categories: (0) in school, (1) CASMIN 1a: inadequately completed, (2) CASMIN 1b: general elementary school, (3) CASMIN 1c: basic vocational qualification, (4) CASMIN 2b: intermediate general qualification, (5) CASMIN 2a: intermediate vocational, (6) CASMIN 2c_gen: general maturity certificate, (7) CASMIN 2c_voc: vocational maturity certificate, (8) CASMIN 3a: lower tertiary education, (9) CASMIN 3b: higher tertiary education.

4 By weighting part-time work experience with a factor of 0.5, we take into account the lower labor-market returns for part-time experience. This is especially relevant for the task at hand because part-time work is more prevalent in western Germany than in eastern Germany (Hobler et al. 2020).

5 We combine some economic areas from the NACE classification with low prevalence. We distinguish between the following economic areas: (1) farming/fishing/mining, (2) manufacturing, (3) electricity/water/waste, (4) construction, (5) wholesale/retail, (6) food/accommodation, (7) transportation/communication, (8) finance/insurance, (9) real estate/business-related services, (10) public administration, (11) education, (12) health/social work, (13) other services.

6 Full replication code is available here: https://osf.io/egpyh

7 We calculated a respondent’s position in the hourly wage distribution for each year and for eastern and western Germany separately. Using region-specific deciles considers that the wage distribution differs by region (Brüll and Gathmann 2020). Figure A2 in the Online Supplement illustrates that our findings are robust to using respondents’ positions in the joint wage distribution instead.
