# Supplementary material for: The East–West Gap in Pay Fairness Perceptions Converged Between 2009 and 2023
Source: Kolner Z Soz Sozpsychol. 2026 Jun 10;78(2):413–29. doi: 10.1007/s11577-026-01086-5 (PMC13309442; doi:10.1007/s11577-026-01086-5)
Supplement: Supplementary file 1 [file 11577_2026_1086_MOESM1_ESM.pdf]

Online Supplement

Table A1 Number of observations, by year

Survey year	N
2009	6,800
2011	7,868
2013	8,606
2015	12,000
2017	12,287
2019	11,141
2021	7,611
2023	9,270
Total	75,583

Data: SOEPv40.

Table A2 Univariate descriptive statistics

	Mean	SD	Min	Max	N
Unfairness of earnings	0.108	0.214	-7.844	2.960	75,583
East Germany (1=yes)	0.170		0	1	75,583
Human capital factors					
Female (1=yes)	0.478		0	1	75,578
No immigration background	0.785		0	1	75,583
Direct immigration background	0.159		0	1	75,583
Indirect immigration background	0.056		0	1	75,583
CASMIN:					
(0) in school	0.001		0	1	73,281
(1a) inadequately completed	0.011		0	1	73,281
(1b) general elementary school	0.047		0	1	73,281
(1c) basic vocational qualification	0.190				
(2b) intermediate general qualification	0.018		0	1	73,281
(2a) intermediate vocational	0.291		0	1	73,281
(2c_gen) general maturity certificate	0.036		0	1	73,281
(2c_voc) vocational maturity certificate	0.134		0	1	73,281
(3a) lower tertiary education	0.105		0	1	73,281
(3b) higher tertiary education	0.167		0	1	73,281
Relative work experience	0.679	0.271	0	7.917	72,211
Age in years	43.39	11.67	18	65	75,583
Structural factors					
Economic area					
Farming/Fishing/Mining	0.012		0	1	69,823
Manufacturing	0.237		0	1	69,823
Electricity/Water/Waste	0.015		0	1	69,823
Construction	0.048		0	1	69,823
Wholesale/Retail	0.111		0	1	69,823
Food/Accommodation	0.026		0	1	69,823
Transportation/Communication	0.074		0	1	69,823
Finance/Insurance	0.035		0	1	69,823
Real Estate/Business-related services	0.094		0	1	69,823
Public administration	0.092		0	1	69,823
Education	0.075		0	1	69,823
Health/Social work	0.146		0	1	69,823
Other services	0.034		0	1	69,823
Public sector (1=yes)	0.270		0	1	75,276
Size of organization					

<20 employees	0.194	0	1	74,993
20 to <200 employees	0.258	0	1	74,993
200 to <2000 employees	0.234	0	1	74,993
>2000 employees	0.313	0	1	74,993

Data: SOEP v40

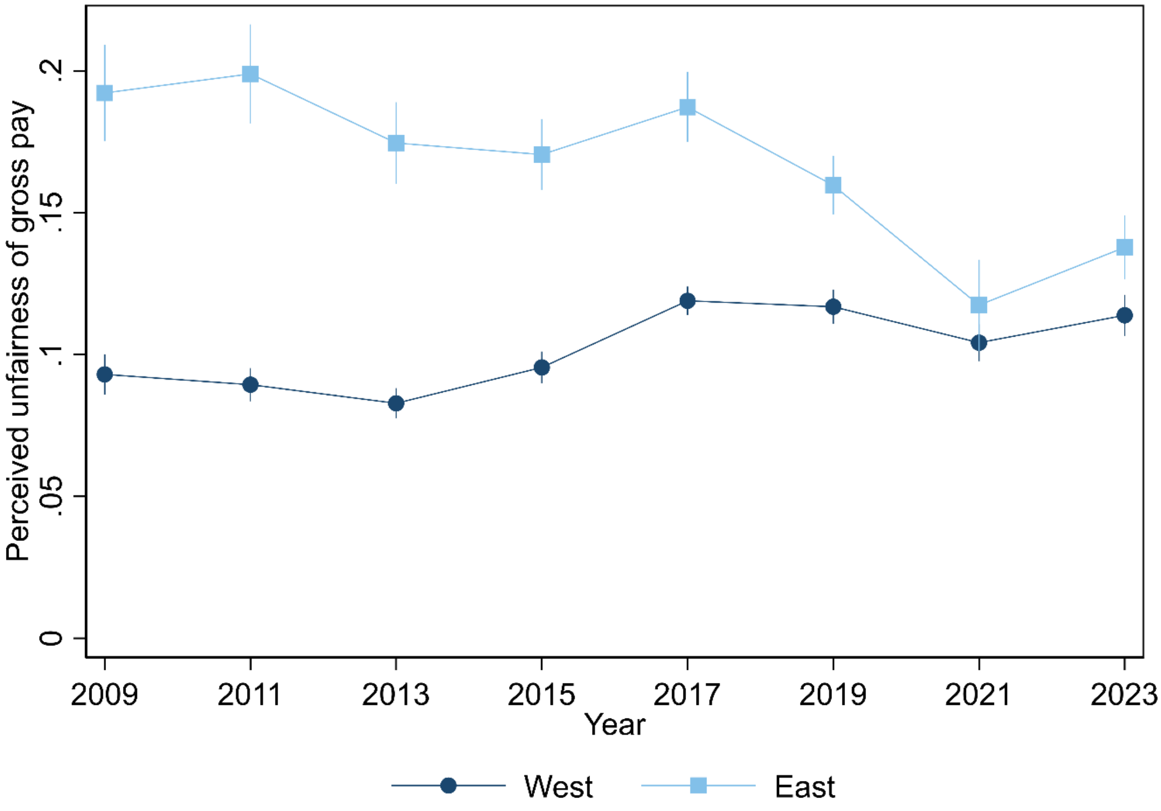

Fig. A1 Trends in perceived unfairness of gross pay among employees in East and West Germany, 2009–2023, including p1 and p99 outliers.

Notes: The figure shows mean values with 95% confidence intervals. A higher value indicates a greater perception of unfair underpayment. In line with Figure 1 of the main document, the data illustrate a gradual convergence between the two regions over time, driven by increasing perceived unfairness in the West and a decline in the East. Data: SOEP v40.

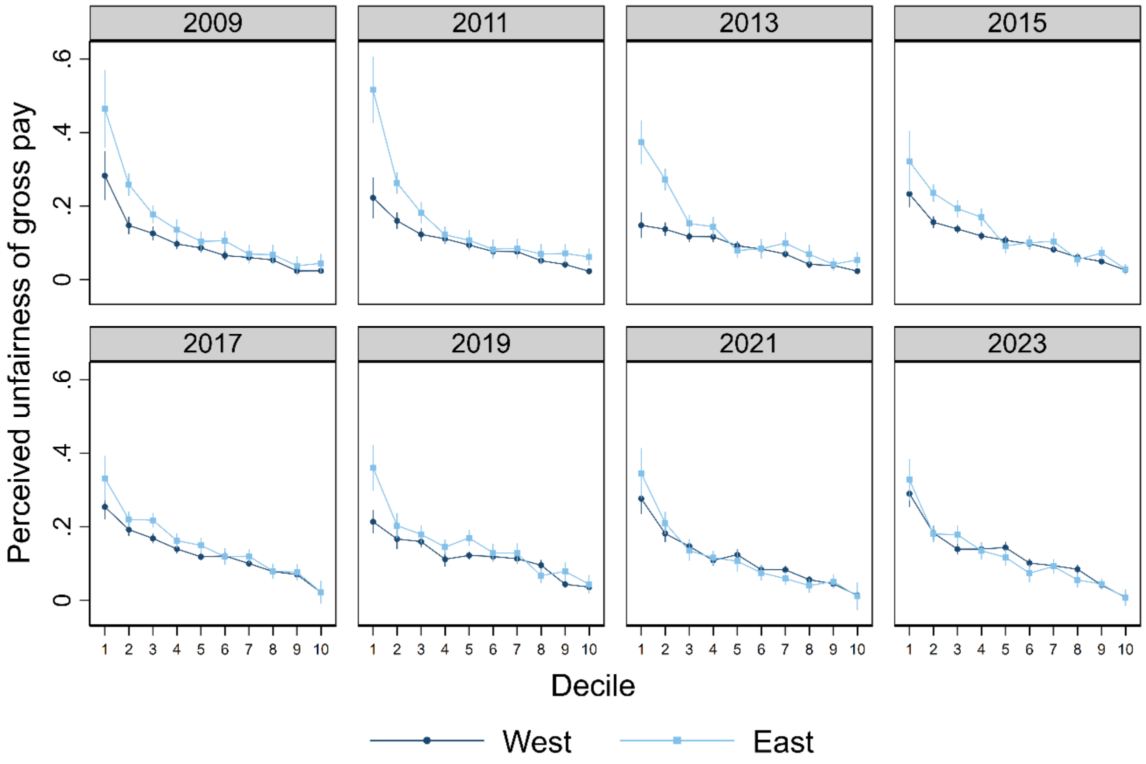

Fig. A2 Perceived unfairness of gross pay among employees in East and West Germany by wage decile, from 2009 to 2023.

Note: In contrast to Figure 2 of the main document, each respondent's position in the wage distribution is calculated based on the joint wage distribution and not separately by region. The overall pattern persists. Regional differences narrowed, driven mainly by declining perceptions of unfairness among lower-wage employees in East Germany. Data: SOEP v40

Table A3 Unfairness of gross pay regressed on regional dummy and covariates

	2009		2011	
	**b**	**SE**	**b**	**SE**
East Germany (1=yes)	0.085***	(0.012)	0.091***	(0.010)
Human capital factors				
Female (1=yes)	-0.018*	(0.009)	-0.014*	(0.007)
No immigration background	0.000	(.)	0.000	(.)
Direct immigration background	0.022	(0.021)	0.018	(0.013)
Indirect immigration background	0.013	(0.017)	0.009	(0.016)
CASMIN:				
(0) in school	0.051	(0.067)	0.054	(0.060)
(1a) inadequately completed	0.002	(0.019)	-0.026	(0.016)
(1b) general elementary school	0.000	(.)	0.000	(.)
(1c) basic vocational qualification	0.014	(0.037)	-0.017	(0.023)
(2b) intermediate general qualification	0.010	(0.011)	0.011	(0.010)
(2a) intermediate vocational	-0.069**	(0.021)	-0.022	(0.026)
(2c_gen) general maturity certificate	-0.003	(0.026)	0.008	(0.019)
(2c_voc) vocational maturity certificate	0.009	(0.026)	0.008	(0.022)
(3a) lower tertiary education	-0.019	(0.044)	0.010	(0.034)
(3b) higher tertiary education	0.005	(0.006)	-0.004	(0.004)
Relative work experience	-0.049	(0.068)	-0.084***	(0.020)
Relative work experience (squared)	-0.008	(0.043)	0.016***	(0.004)
Age in years	-0.005	(0.003)	0.000	(0.002)
Age in years (squared)	0.000	(0.000)	0.000	(0.000)
Structural factors				
Economic area				
Farming/Fishing/Mining	Ref.		Ref.	
Manufacturing	-0.107*	(0.052)	-0.090	(0.056)
Electricity/Water/Waste	-0.173**	(0.054)	-0.128*	(0.059)
Construction	-0.127*	(0.054)	-0.113*	(0.057)
Wholesale/Retail	-0.082	(0.054)	-0.075	(0.056)
Food/Accommodation	-0.033	(0.068)	-0.086	(0.060)
Transportation/Communication	-0.088	(0.053)	-0.055	(0.060)
Finance/Insurance	-0.131*	(0.054)	-0.099	(0.057)
Real Estate/Business-related services	-0.085	(0.054)	-0.075	(0.057)
Public administration	-0.128*	(0.054)	-0.110	(0.059)
Education	-0.100	(0.056)	-0.053	(0.060)
Health/Social work	-0.072	(0.054)	-0.051	(0.057)
Other services	-0.043	(0.059)	-0.031	(0.062)
Public sector (1=yes)	-0.003	(0.011)	-0.020	(0.012)
Size of organization				
<20 employees	Ref.		Ref.	
20 to <200 employees	-0.018	(0.012)	-0.019	(0.011)
200 to <2000 employees	-0.025*	(0.012)	-0.031**	(0.011)
>2000 employees	-0.039**	(0.013)	-0.048***	(0.012)
Constant	0.296**	(0.102)	0.295**	(0.096)
N	6468		7409	
R^2	0.059		0.064	

Table A3 Unfairness of gross pay regressed on regional dummy and covariates (continued)

	2013		2015	
	**b**	**SE**	**b**	**SE**
East Germany (1=yes)	0.072***	(0.009)	0.053***	(0.008)
Human capital factors				
Female (1=yes)	-0.008	(0.006)	-0.008	(0.006)
No immigration background	Ref.		Ref.	
Direct immigration background	-0.042***	(0.008)	0.001	(0.008)
Indirect immigration background	-0.033**	(0.011)	0.003	(0.013)
CASMIN:				
(1a) inadequately completed	-0.042	(0.047)	0.050	(0.032)
(1b) general elementary school	-0.033	(0.042)	0.005	(0.024)
(1c) basic vocational qualification	-0.052	(0.036)	-0.005	(0.021)
(2b) intermediate general qualification	Ref.		Ref.	
(2a) intermediate vocational	-0.061	(0.036)	0.006	(0.020)
(2c_gen) general maturity certificate	-0.069	(0.039)	-0.017	(0.024)
(2c_voc) vocational maturity certificate	-0.061	(0.037)	-0.018	(0.021)
(3a) lower tertiary education	-0.059	(0.036)	-0.009	(0.022)
(3b) higher tertiary education	-0.072*	(0.037)	-0.025	(0.020)
Relative work experience	-0.063*	(0.025)	-0.038*	(0.018)
Relative work experience (squared)	0.006	(0.009)	0.013**	(0.004)
Age in years	-0.001	(0.002)	0.002	(0.002)
Age in years (squared)	0.000	(0.000)	-0.000	(0.000)
Structural factors				
Economic area				
Farming/Fishing/Mining	Ref.		Ref.	
Manufacturing	-0.068	(0.039)	-0.021	(0.032)
Electricity/Water/Waste	-0.109**	(0.041)	-0.039	(0.037)
Construction	-0.044	(0.041)	0.016	(0.034)
Wholesale/Retail	-0.035	(0.040)	0.006	(0.033)
Food/Accommodation	-0.061	(0.042)	0.035	(0.038)
Transportation/Communication	-0.032	(0.041)	0.016	(0.034)
Finance/Insurance	-0.077	(0.041)	-0.031	(0.033)
Real Estate/Business-related services	-0.055	(0.040)	-0.013	(0.032)
Public administration	-0.074	(0.041)	-0.026	(0.033)
Education	-0.043	(0.042)	-0.003	(0.033)
Health/Social work	-0.017	(0.041)	0.036	(0.032)
Other services	-0.031	(0.043)	-0.000	(0.035)
Public sector (1=yes)	-0.014	(0.011)	-0.009	(0.009)
Size of organization				
<20 employees	Ref.		Ref.	
20 to <200 employees	0.002	(0.010)	0.006	(0.009)
200 to <2000 employees	-0.009	(0.010)	-0.017	(0.010)
>2000 employees	-0.029**	(0.009)	-0.040***	(0.009)
Constant	0.260***	(0.067)	0.115*	(0.053)
N	8,082		11,355	
R^2	0.066		0.050	

Table A3 Unfairness of gross pay regressed on regional dummy and covariates (continued)

	2017		2019	
	**b**	**SE**	**b**	**SE**
East Germany (1=yes)	0.049***	(0.007)	0.051***	(0.010)
Human capital factors				
Female (1=yes)	-0.020**	(0.006)	-0.026**	(0.008)
No immigration background	Ref.		Ref.	
Direct immigration background	0.014	(0.008)	0.014	(0.011)
Indirect immigration background	0.005	(0.015)	-0.001	(0.017)
CASMIN:				
(1a) inadequately completed	0.005	(0.030)	0.042	(0.039)
(1b) general elementary school	-0.007	(0.027)	-0.012	(0.030)
(1c) basic vocational qualification	-0.005	(0.023)	-0.006	(0.027)
(2b) intermediate general qualification	Ref.		Ref.	
(2a) intermediate vocational	0.006	(0.023)	-0.009	(0.028)
(2c_gen) general maturity certificate	-0.027	(0.027)	-0.031	(0.049)
(2c_voc) vocational maturity certificate	0.004	(0.023)	0.010	(0.028)
(3a) lower tertiary education	-0.005	(0.024)	0.001	(0.028)
(3b) higher tertiary education	-0.025	(0.023)	-0.020	(0.027)
Relative work experience	-0.042*	(0.020)	-0.015	(0.022)
Relative work experience (squared)	0.009	(0.006)	-0.001	(0.004)
Age in years	0.001	(0.002)	0.003	(0.002)
Age in years (squared)	-0.000	(0.000)	-0.000	(0.000)
Structural factors				
Economic area				
Farming/Fishing/Mining	Ref.		Ref.	
Manufacturing	-0.041	(0.021)	-0.036	(0.042)
Electricity/Water/Waste	-0.054*	(0.027)	-0.031	(0.044)
Construction	-0.016	(0.024)	0.012	(0.048)
Wholesale/Retail	0.003	(0.022)	0.016	(0.043)
Food/Accommodation	0.023	(0.028)	0.035	(0.047)
Transportation/Communication	0.003	(0.024)	0.004	(0.043)
Finance/Insurance	-0.054*	(0.024)	-0.032	(0.044)
Real Estate/Business-related services	-0.026	(0.022)	-0.005	(0.043)
Public administration	-0.047	(0.025)	-0.014	(0.043)
Education	0.006	(0.025)	0.003	(0.043)
Health/Social work	0.013	(0.023)	0.043	(0.043)
Other services	-0.010	(0.025)	0.025	(0.045)
Public sector (1=yes)	-0.009	(0.010)	0.001	(0.012)
Size of organization				
<20 employees	Ref.		Ref.	
20 to <200 employees	0.005	(0.009)	-0.004	(0.010)
200 to <2000 employees	-0.014	(0.009)	-0.010	(0.014)
>2000 employees	-0.021*	(0.009)	-0.034**	(0.011)
Constant	0.168***	(0.049)	0.104	(0.073)
N	11,586		10,415	
R^2	0.034		0.028	

Table A3 Unfairness of gross pay regressed on regional dummy and covariates (continued)

	2021		2023	
	**b**	**SE**	**b**	**SE**
East Germany (1=yes)	0.025*	(0.010)	0.027*	(0.013)
Human capital factors				
Female (1=yes)	0.006	(0.009)	-0.005	(0.010)
No immigration background	Ref.		Ref.	
Direct immigration background	0.015	(0.013)	0.017	(0.016)
Indirect immigration background	0.014	(0.020)	0.016	(0.020)
CASMIN:				
(1a) inadequately completed	-0.014	(0.054)	-0.018	(0.071)
(1b) general elementary school	-0.044	(0.038)	-0.054	(0.048)
(1c) basic vocational qualification	-0.027	(0.035)	-0.047	(0.041)
(2b) intermediate general qualification	Ref.		Ref.	
(2a) intermediate vocational	-0.015	(0.035)	-0.065	(0.039)
(2c_gen) general maturity certificate	-0.088*	(0.040)	-0.104*	(0.044)
(2c_voc) vocational maturity certificate	-0.037	(0.034)	-0.062	(0.040)
(3a) lower tertiary education	-0.033	(0.037)	-0.050	(0.041)
(3b) higher tertiary education	-0.036	(0.035)	-0.074	(0.039)
Relative work experience	-0.088**	(0.032)	-0.008	(0.036)
Relative work experience (squared)	0.027**	(0.008)	-0.002	(0.013)
Age in years	-0.012***	(0.003)	-0.008	(0.004)
Age in years (squared)	0.000**	(0.000)	0.000	(0.000)
Structural factors				
Economic area				
Farming/Fishing/Mining	Ref.		Ref.	
Manufacturing	0.056	(0.077)	-0.038	(0.050)
Electricity/Water/Waste	0.050	(0.079)	-0.057	(0.055)
Construction	0.045	(0.078)	-0.036	(0.052)
Wholesale/Retail	0.090	(0.077)	0.007	(0.052)
Food/Accommodation	0.182	(0.103)	-0.029	(0.058)
Transportation/Communication	0.075	(0.077)	-0.010	(0.051)
Finance/Insurance	0.030	(0.077)	-0.069	(0.050)
Real Estate/Business-related services	0.066	(0.077)	-0.010	(0.051)
Public administration	0.047	(0.077)	-0.029	(0.050)
Education	0.089	(0.078)	0.000	(0.050)
Health/Social work	0.091	(0.076)	0.028	(0.050)
Other services	0.110	(0.082)	0.001	(0.052)
Public sector (1=yes)	-0.004	(0.012)	-0.017	(0.016)
Size of organization				
<20 employees	Ref.		Ref.	
20 to <200 employees	-0.004	(0.018)	-0.010	(0.018)
200 to <2000 employees	-0.013	(0.018)	-0.023	(0.019)
>2000 employees	-0.043*	(0.018)	-0.015	(0.018)
Constant	0.389***	(0.111)	0.382***	(0.098)
N	6,022		5,263	
R^2	0.058		0.032	

Robust standard errors in parentheses. * $p < 0.05$, ** $p < 0.010$, *** $p < 0.001$. *Data: SOEPv40.*

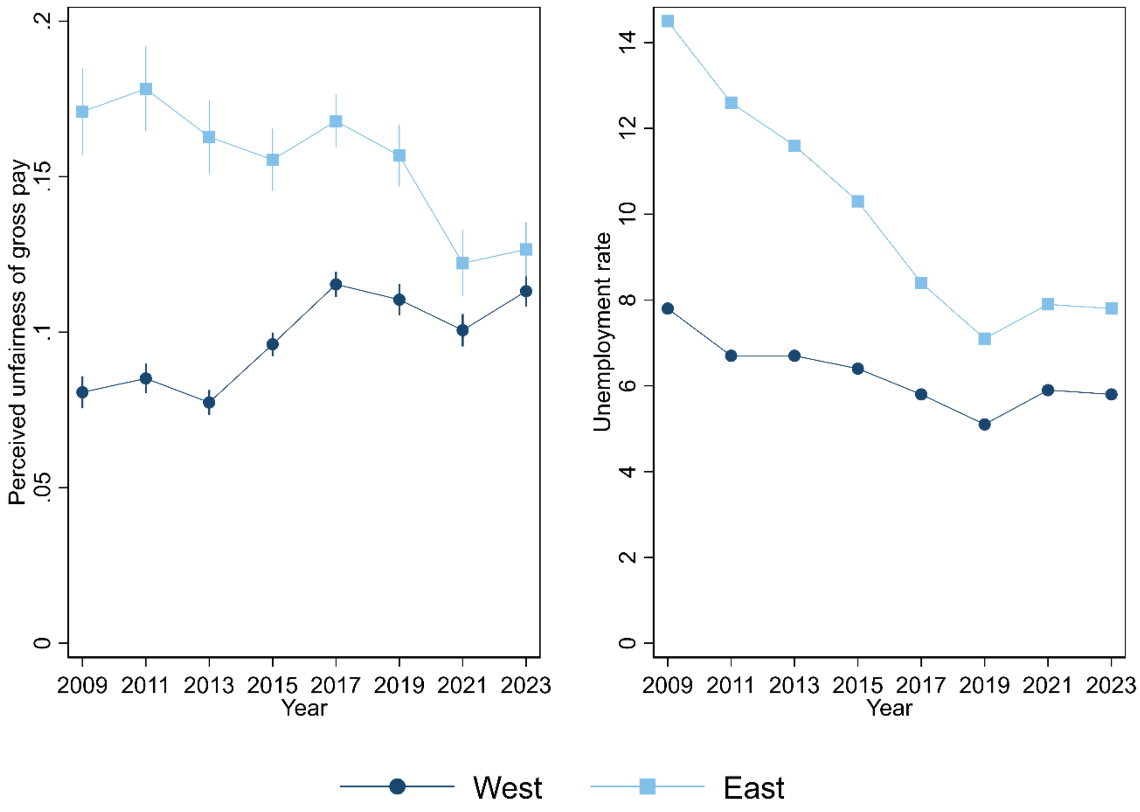

Fig. A3 Trends in perceived unfairness of gross pay among employees in East and West Germany, 2009–2023, and simultaneously trend in unemployment.

Notes: The left panel is identical to Figure 1 of the main analysis and illustrates the gradual convergence between the two regions over time, driven by increasing perceived unfairness in the West and a decline in the East. The panel on the right maps over regions-specific unemployment rate over the same period of time. For East Germany, there is some overlap in the general trend between unemployment and decreasing fairness perceptions, but in West Germany, the trends in unemployment and unfairness seem completely decoupled. Data: SOEP v40.
